# Severe Preeclampsia in the Setting of Myasthenia Gravis

**DOI:** 10.1155/2017/9204930

**Published:** 2017-02-09

**Authors:** Adam J. Lake, Antoun Al Khabbaz, Renée Keeney

**Affiliations:** ^1^Department of Obstetrics and Gynecology, University of Illinois College of Medicine at Rockford, 1601 Parkview Ave., Rockford, IL 61101, USA; ^2^Department of Women, Children, and Family Health, University of Illinois College of Nursing at Rockford, 1601 Parkview Ave., Rockford, IL 61101, USA

## Abstract

Myasthenia gravis (MG) is a rare autoimmune disease that leads to progressive muscle weakness and is common during female reproductive years. The myasthenic mother and her newborn must be observed carefully, as complications during all stages of pregnancy and the puerperium may arise suddenly. Preeclampsia is a common obstetrical condition for which magnesium sulfate is used for seizure prophylaxis. However, magnesium sulfate is strongly contraindicated in MG as it impairs already slowed nerve-muscle connections. Similarly, many first-line antihypertensive medications, including calcium channels blockers and *β*-blockers, may lead to MG exacerbation. This case describes the effective obstetrical management of a patient with MG who developed severe preeclampsia. The effective use of levetiracetam and various antihypertensive medications including intravenous labetalol is described. A review of the ten reported cases of MG complicated by preeclampsia is examined to aggregate observations of clinical care, with focus on delivery methods, anticonvulsants, and antihypertensive medications.

## 1. Introduction

Myasthenia gravis (MG) is an autoimmune disease in which antibodies most frequently target muscle nicotinic acetylcholine receptors (nAChR) or muscle-specific kinase (MuSK), which leads to the gradual attrition of neuromuscular signals. This manifests itself as fatigue and progressive paresis of skeletal muscle, which characteristically worsens with exertion and improves with rest. There is an estimated MG prevalence of 1 per 5,000 individuals in the United States [1] with maternal MG complicating 1 in 68,000 pregnancies [2]. Exacerbations of MG are termed myasthenic crises and are often precipitated by infections, antibiotics, emotional stress, and surgery [3]. A myasthenic crisis may lead to life-threatening acute respiratory failure requiring mechanical ventilation. With improved neurocritical care protocols, mortality from a myasthenic crisis has improved to 5% [3]. The Myasthenia Gravis Foundation of America (MGFA) treatment guidelines recommend the use of acetylcholinesterase inhibitors, intravenous immunoglobulins, plasma exchange (PLEX), glucocorticoids, and thymectomy for myasthenia treatment [4].

Preeclampsia is a systemic disorder characterized by new-onset hypertension, proteinuria, and end-organ damage after 20-week gestation and complicates 2–8% of pregnancies in the United States [5]. Treatment of hypertension in the setting of preeclampsia may be approached aggressively with multiple antihypertensive medications to achieve adequate blood pressure control. Magnesium sulfate, shown to be superior to other anticonvulsants, is used frequently for seizure prophylaxis [6]. Both MG and preeclampsia have specific treatment guidelines, which are often sufficient for adequate control of each disease. However, management of preeclampsia with magnesium sulfate and commonly used antihypertensive medications, such as *β*-blockers and calcium channel blockers, is contraindicated in MG as it may exacerbate MG symptomatology and precipitate a myasthenic crisis. An association between MG and preeclampsia prevalence has not been demonstrated in the English literature. There is a paucity of reports describing treatment of preeclampsia in patients with MG.

We present a pregnancy complicated by preexisting MG and the later development of severe preeclampsia with description of novel clinical management with intravenous levetiracetam and labetalol. A review of the English literature is presented as well, describing experiences with this rare clinical scenario.

## 2. Presentation of Case

A 28-year-old G3P2002 patient at 34-week gestation was admitted to the labor and delivery suite with a diagnosis of preeclampsia. The patient had two prior uncomplicated spontaneous vaginal deliveries. Her pregnancy was dated by ultrasound at 8-week gestation. The patient is known to have MG managed with pyridostigmine 30.0 mg orally, three times a day. She had a thymectomy six years prior to this pregnancy. She had an uncomplicated prenatal course. Her blood pressure during pregnancy ranged from 108 to 132 mmHg systolic and 67 to 88 mmHg diastolic. She has a history of preexisting prehypertension and was on no antihypertensive treatment. Upon arrival to the hospital, the patient's initial blood pressure was in the range of 170–180 mmHg systolic and 100–110 mmHg diastolic. She was in no acute distress and reported no clinically significant edema, right upper quadrant pain, weakness, dyspnea, diplopia, or ptosis. Her only notable symptom was a new-onset, mild headache. She denied experiencing contractions, leakage of fluid per vagina, or vaginal bleeding, and she reported normal fetal movements. Workup for preeclampsia showed an elevated protein/creatinine ratio of 0.7, a slightly elevated uric acid of 5.6 mg/dL, creatinine of 0.74 mg/dL, normal liver enzymes (AST 26 U/L, ALT 11 U/L), and platelet count 152 × 10^3^/*μ*L.

She was started on intravenous levetiracetam (1.0 g intravenous bolus for seizure prophylaxis). She was given multiple doses of 5.0 mg intravenous hydralazine to treat her hypertension, which had minimal effect. Her blood pressure was as high as 229/117 mmHg. The patient was then given labetalol intravenously resulting in better control of her blood pressure with no MG exacerbation noted. Within a few hours of admission, the patient developed mild clonus and her headache increased in severity. The patient's cervix at this point was unfavorable so the decision was taken to proceed with Cesarean section under spinal anesthesia. Surgery was performed without complications, resulting in the birth of a healthy male newborn with a birth weight of 1865 g and APGAR scores of 8 at one minute and 9 at five minutes. The newborn was followed up closely and showed no signs of transient neonatal MG.

In the postpartum period, levetiracetam was continued at a dose of 500 mg intravenously every 12 hours and was discontinued 2 days postpartum. The only notable side effects were somnolence and fatigue. These symptoms resolved after discontinuation of levetiracetam. Hypertension was treated with methyldopa and hydralazine. Clonidine was added on postpartum day 2. The patient was discharged home on postpartum day 4 on methyldopa and clonidine in stable condition with blood pressure in the prehypertensive range. The patient had no myasthenic crisis in either the intrapartum or the postpartum periods. She was weaned off her antihypertensive medications postpartum and was normotensive at her six-week postpartum visit.

## 3. Discussion

MG exacerbations are common in pregnancy, perhaps due to increases in blood volume and changes in the renal clearance of medications [17]. MG is associated with other autoimmune diseases such as rheumatoid arthritis and thyroid dysfunction but is not related to any increase in the prevalence of preeclampsia [18, 19]. The clinical course of MG in pregnancy is variable, with an improvement of symptoms in 29% of pregnancies, worsening in 41%, and no change in 30% [20]. Pregnant patients with MG must be watched closely due to its unpredictable course in pregnancy. Chorioamnionitis, postpartum endometritis, and mastitis should be treated effectively as they can precipitate a myasthenic crisis [17]. Ideally, patients with MG should have optimization of their myasthenic symptoms prior to attempting to conceive [4]. Transient neonatal myasthenia gravis is due to transplacental passage of maternal IgG autoantibodies [17] and presents with mask-like facies, a weakened cry, and impaired ability to suck. Clinicians recommend neonatal observation for a minimum of 2 days prior to discharge [4, 21]. Breastfeeding is not contraindicated in MG unless the patient is in a myasthenic crisis [15].

A comprehensive literature review of published cases of MG and preeclampsia in the English literature published from 1976 to 2016 using a MEDLINE search is presented (Table 1). Ten case reports have been published. A variety of medications have been implicated in inducing myasthenic crises and should be avoided in all MG patients (Table 2) [17, 22]. Paradoxically, this includes the initiation of corticosteroid treatment, which causes exacerbations in 50% of patients [3]. These cases provide insight into the modes of delivery, anticonvulsants, and antihypertensive medications used for this clinical situation.

Delivery was accomplished by vaginal delivery in four of the reported cases; the other six patients delivered by Cesarean section. Surgical delivery may lead to exacerbations of MG and should only be performed for maternal or fetal indications [11]. For this case and previous cases, Cesarean sections were considered beneficial due to concerns of worsening maternal status from preeclampsia [12] with consideration taken that surgery itself could cause a myasthenic crisis. Regional anesthesia is preferred over general anesthesia for both vaginal and surgical deliveries. Of the reported cases, seven patients received regional anesthesia. One of those patients had profound vagal bradycardia following neuraxial anesthetic blockage, which required atropine and ephedrine to resolve [14]. General anesthesia with intubation was performed in only one case, and prolonged ventilation was needed postpartum. In that case, the muscle relaxant pancuronium was used. Muscle relaxants, both depolarizing and nondepolarizing, are also to be avoided as MG patients are quite sensitive to their effects [21].

Our patient received spinal anesthesia for her Cesarean section due to concerns of worsening severe preeclampsia. The surgery was uncomplicated and the patient had no residual side effects from the anesthesia.

Calcium channel blockers and *β*-blockers can exacerbate MG symptoms [14]. Hence, their use should be avoided if possible for a patient with MG. Methyldopa and hydralazine are considered safe to use in MG patients. Severe hypertension (≥160/110 mmHg) should be treated aggressively to prevent stroke and intracranial bleeding. Intravenous labetalol is widely used for treatment of severe hypertension in the intrapartum and postpartum periods. However, its use can cause weakness in MG patients. As such, patients should be monitored closely due to potential worsening of weakness [14]. A labetalol intravenous drip was effective in controlling severe hypertension in one reported patient with MG and preeclampsia [13]. The patient had transient weakness without any other significant neurologic side effects. Labetalol use should be restricted to cases with poor blood pressure control with hydralazine, and close monitoring of MG symptoms should be implemented.

Our patient was treated first with multiple doses of 5.0 mg intravenous hydralazine but her blood pressure remained as high as 229/117 mmHg. Labetalol 20.0 mg intravenously was given, leading to adequate blood pressure control with no worsening fatigue, weakness, or other symptoms of MG. A total of three doses of labetalol intravenously were given.

The American Congress of Obstetrics and Gynecology recommends the use of magnesium sulfate for seizure prophylaxis in preeclampsia; however, magnesium sulfate is strongly contraindicated in MG as it impairs already slowed nerve-muscle contractions through a competitive mechanism involving calcium at the neuromuscular junction [23]. Serum magnesium concentrations cause typical, dose-dependent side effects such as fatigue, facial flushing, arrhythmias, and muscle weakness. In neuromuscular disease, symptoms develop at lower magnesium levels [9]. The first reported case of MG with preeclampsia was treated with two intramuscular doses of 5.0 g of magnesium sulfate [6]. The patient developed weakness and profound obtundation followed by sudden acute respiratory insufficiency. Of the ten previously reported cases, anticonvulsant treatment has been reported in three cases. Two patients were treated with magnesium sulfate and one patient was treated with diazepam. Both patients treated with magnesium sulfate had a myasthenic crisis; one of them developed transient quadriplegia [7, 8, 10].

Few studies suggest phenytoin and diazepam as an alternative for seizure prophylaxis in preeclampsia. Systematic reviews concluded that they are less effective than magnesium sulfate in reducing mortality [18, 24]. A UK multispecialty group recommended magnesium sulfate use only in those women who experience eclamptic seizures, with the caveat that mechanical ventilation should be prepared and an obstetrical anesthesiologist and neurologist should be on site [21].

Our patient was treated with levetiracetam for seizure prophylaxis. The patient did well with no significant neurological symptoms, eclamptic seizures, or myasthenic symptoms. Levetiracetam is a relatively safe (Pregnancy Risk Category C) antiepileptic medication. Advantages for use in pregnancy include a lower need for drug level monitoring and fewer interactions with other medications. A recent systematic review showed no significant increase in major congenital malformations compared with unexposed controls [25].

Obstetrical care of the myasthenic patient is ideally a team-based approach involving obstetricians, neurologists, anesthesiologists, and neonatologists. The pregnant patient with MG and preeclampsia should be hospitalized with any sign of elevated blood pressure. Cesarean section with spinal anesthesia should be performed only for obstetrical indications as it may precipitate a myasthenic crisis but has generally good outcomes. Hydralazine is considered the drug of choice for treatment of severe preeclampsia, while intravenous labetalol can be used for refractory hypertension but requires close monitoring of the patient. Levetiracetam can potentially be used effectively and safely for seizure prophylaxis in this rare clinical scenario.

## Figures and Tables

**(a):** 

Author(s)	Mother's age, gravidity and parity, and gestational age at admission	Mode of delivery	Anesthesia	Hypertensive treatment	Anticonvulsant treatment	Complications
Cohen et al. [7] (1976)	37 yo, G3P1, term	Spontaneous vaginal delivery	Spinal anesthesia	Furosemide and methydopa	Magnesium sulfate (IM injection)	Within 10 minutes of IM magnesium sulfate, the patient had a myasthenic crisis but improved quickly with 1.0 g calcium gluconate, 0.4 mg atropine, and 10.0 mg edrophonium.
Duff [8] (1979)	26 yo, G2P1, 37 weeks	Cesarean section	General anesthesia (thiopentone, scoline, pancuronium, and nitrous oxide)	Poor control with methyldopa, diazoxide, reserpine, and furosemide	Diazepam “for sedation”	The patient required 16 hours of ventilator support because of respiratory insufficiency after general anesthesia.
Duff (1979)	36 yo, G2P1, 36 weeks	Vaginal delivery after labor induction	Spinal anesthesia	Ephedrine (no evidence for use currently)	None reported	MG did not improve in the postpartum period, so the patient was started on prednisone. The patient was discharged five weeks postpartum with improvement of MG symptoms.
Brogan and Corcoran [9] (1983)	37 yo, G1P0, 32 weeks	Cesarean section	Spinal anesthesia	None reported	None reported	No complications noted.
Bashuk and Krendel [10] (1990)	19 yo, G1P0, term	Spontaneous vaginal delivery	None noted	None reported	Magnesium sulfate IV (4.0 g once) and IM (5.0 g q4h)	Weakness worsened with each IM injection and she became quadriplegic. Once treatment was stopped, she regained muscle strength within a day.

**(b):** 

Author(s)	Mother's age, gravidity and parity, and gestational age at admission	Mode of delivery	Anesthesia	Hypertensive treatment	Anticonvulsant treatment	Clinical presentation
Benshushan et al. [11] (1994)	31 yo, G1P0, 31 weeks	Vaginal delivery after labor induction and artificial rupture of membranes	None noted	Methyldopa and hydralazine, later using furosemide with little diuresis	None reported	Delivery complicated by hemorrhage. The patient was transferred to the ICU for dyspnea, oliguria, and weakness where treatment was started with dopamine, furosemide, IV corticosteroids, and IV pyridostigmine. The patient was discharged ten days later.
Di Spiezio Sardo et al. [12]	27 yo, G2P0, 37 weeks	Cesarean section	Spinal anesthesia	Methyldopa	None reported	The patient was diagnosed with severe preeclampsia complicated by HELLP syndrome. The patient was discharged on day six postpartum.
Hamaoui and Mercado [13] (2009)	31 yo, G7P3, 27 weeks	Cesarean section	Spinal anesthesia (bupivacaine)	Hydralazine, metoprolol, losartan, amlodipine, and labetaol. Labetalol drip controlled BP.	None reported	Three days postpartum, the patient developed HELLP syndrome and myasthenic exacerbation.
Ozcan et al. [14] (2015)	34 yo, G1P0, 36 weeks	Cesarean section	Spinal anesthesia (bupivacaine and fentanyl)	Enalapril	None reported	The patient became bradycardic (42 bpm) after spinal anesthesia, requiring atropine and ephedrine to resolve. Postpartum period required enalapril, with increased dose of pyridostigmine and IV immunoglobulin to resolve symptoms. The patient was discharged 8 days after the Cesarean section.
Sikka et al. [15] (2015)	25 yo, G1P0, 36 weeks	Cesarean section	Spinal anesthesia	None reported	None reported	No complications noted.

**Table 2:** Medications with associated exacerbations of myasthenia gravis.

Magnesium salts	Magnesium sulfate and milk of magnesia, magnesium in multivitamins acceptable
Calcium channel blockers	Amlodipine, nifedipine, and verapamil
*β*-Blockers	Atenolol, propranolol, timolol (including eye drops), and nadolol
Aminoglycosides	Gentamycin, clindamycin, streptomycin, and to lesser extent tobramycin
Macrolides	Azithromycin and erythromycin
Fluoroquinolones	Ciprofloxacin and levofloxacin
D-Penicillamine	Strong association with worsening symptoms of myasthenia, considered contraindicated
Psychiatric medications	TCAs, haloperidol, and lithium
Steroids	Often used in myasthenia treatment, causing transient worsening of symptoms
Statins [16]	Atorvastatin, simvastatin, rosuvastatin, lovastatin, and pravastatin
